# Supplementary material for: PON1 Q192R is associated with high platelet reactivity with clopidogrel in patients undergoing elective neurointervention: A prospective single-center cohort study
Source: PLoS One. 2021 Aug 5;16(8):e0254067. doi: 10.1371/journal.pone.0254067 (PMC8341610; doi:10.1371/journal.pone.0254067)
Supplement: Attachment — Submitted filename: Response to reviewers.docx [file pone.0254067.s002.docx]

PONE-D-21-03186
*PON1* Q192R is associated with high platelet reactivity with clopidogrel in patients undergoing elective neurointervention: a prospective single-center cohort study

Thank you for your comments. We have revised the manuscript and provided point-by-point responses to your comments below. Changes in the manuscript are highlighted in yellow. We hope that our responses are suitable.

Reply to Reviewer #1:

Major comments:

1. **The authors have not included in the analysis other genes and polymorphisms that could be of interest, so this study has a very large limitation in terms of results.**

We fully agree with your comment. Because of the study design, we could not investigate genetic polymorphisms other than *CYP2C19*2*, **3*, **17*, and *PON1* Q192R. Therefore, this study could not exclude alternative results that might involve other genetic polymorphisms. This is one of the major limitations of this study. We have added the words “this study has not included genetic polymorphisms other than *CYP2C19*2*, **3*, and **17*, and *PON1* Q192R on post-clopidogrel platelet reactivity” (page 19, lines 287–289) and “Therefore, this study cannot exclude alternative results that might be related to other genetic polymorphisms.” (page 19, lines 292–293) as the first limitation in the Discussion. We have also added “, although alternative results related to other genetic polymorphisms cannot be excluded” in the Conclusions in the Abstract (page 3, lines 62–63) and the main body of text (page 20, lines 307–308).

2. **The authors should analyze at least the PON1 L55M polymorphism which reduces enzyme activity and is present in 5% of the Japanese population.**

Thank you for your comment. As you mentioned, *PON1* L55M (rs854560; c.163T>A or p.Leu55Met) was also reported as a determinant for the activity of PON1 (Brophy VH, et al. Am J Hum Genet 2001;68:1428-1436). However, we did not focus on *PON1* L55M because to the best of our knowledge, no previous report has demonstrated a direct association between *PON1* L55M and clopidogrel efficacy. In relation to comment #1, we have added reference 34 and the following sentence “Notably, PON1 L55M, which lowers the activity of PON1 [34], and has an allele frequency of 3.4% in East Asians [21], was not included in the analysis” to the Discussion (page 19, lines 289–290).

3. **The result they present is an influence of the 192R allele on platelet aggregation, showing a worse response to clopidogrel in patients who carry it. The study by Bouman et al (ref 8) finds the opposite, but the authors do not mention it in the discussion.**

As you mentioned, Bouman et al. reported a significant influence of the *PON1* 192Q allele on platelet aggregation. Conversely, recent reports (references 13–16) and this study demonstrated the opposite result. This indicates a difference in the role of PON1 between Western and East Asian populations. PON1 contributes to the antioxidative function of high-density lipoprotein-cholesterol, which potentially suppresses platelet aggregation (Mackness MI et al. Atherosclerosis 1993;104:129–135.). Therefore, in addition to *CYP2C19* and *PON1* polymorphisms, differences in lipoprotein levels and their association with vascular disease risk (Paré G, et al. Circulation. 2019;139:1472-1482.) might explain these results. We have added references 30 and 31 and new text to the Discussion (page 18, lines 257–262).

4. **It is important to note that 85% of the prodrug is metabolized by CES1 and only the remaining 15% will be metabolized to form the active metabolite, with CYP2C19 being responsible for the formation of 45% of 2-oxo-clopidogrel and 21% of the active metabolite. Bearing in mind that many other enzymes are also involved, the role of PON1 could appear to be minor. The authors do not comment on this.**

We appreciate your comment. As you mentioned, many enzymes other than CYP2C19 and PON1 are also involved in the metabolism of clopidogrel, and therefore, the role of PON1 might be relatively minor. We have changed reference 31 to reference 27 and added the words “About 85% of clopidogrel is metabolized by CES1 and the remaining 15% is” (page 17, lines 235–236), “—the formation of 45% of 2-oxo-clopidogrel and 21% of the active metabolite [27]—” (page 17, line 241), and “Moreover, in addition to the partial role of CYP2C19, many other enzymes are involved in the metabolism of clopidogrel, and the role of PON1 could be relatively minor.” (page 19, lines 290–292) in the Discussion.

5. **The authors do not comment on the discussion of the only article that analyzes PON1 in neurointerventional patients, also analyzing many more genes and polymorphisms (Saiz-Rodríguez et al, ref 18). In that article no association with PON1 was found.**

Although no significant association between PON1 polymorphisms and response to clopidogrel was reported by Saiz-Rodríguez et al., this might be due to ethnic differences or combined assessment of the PON1 polymorphisms, *PON1* Q192R, L55M (rs854560; c.163T>A or p.Leu55Met), and C-108T (rs705379; c.-108C>T). We have added a sentence to the Discussion mentioning this previous study (page 17, line 253 – page 18, line 257).

6. **Authors state that they include the use of proton pump inhibitors, almost 75% of patients were receiving a PPI, which have been highly linked to clopidogrel response, via inhibition of CYP2C19 (Saiz-Rodríguez et al, ref 18), Saab et al. Ther Clin Risk Manag. 2015 Sep 23;11:1421-7, but they found no association? It’s doubtful.**

Thank you for your comment. Although previous reports showed a significant association between proton pump inhibitors (PPI) and attenuated clopidogrel efficacy, it was inconclusive, especially in East Asians (Zou D, et al. J Gastroentenol Hepatol 2017;32:1152-1159). Our study results were similar to those reported by Lin et al., in which no robust interaction was found between PPI and clopidogrel efficacy evaluated by the VerifyNow P2Y12 assay (Lin SF, et al. Medicine 2020;99:50).

**Did the authors applied any multiple test correction?**

In this study, we did not correct for multiple comparisons, which could have led to false-positive results. We have added the words “Fourth, the lack of multiple-comparisons correction might have led to false-positive results” to the Discussion (page 19, line 300 – page 20, line 301).

Minor comments:

1. **Loss-of-function and gain –of-function terminology is obsolete. Please change accordingly throughout the manuscript based on the CPIC standardization of terms (Caudle et al.)**

Thank you for your comment. According to your suggestion, we changed the descriptions of *CYP2C19*2* and **3* to “no function”, and **17* to “increased function”, throughout the revised manuscript.

2. **Enrollment finished in 2015, why? Can authors enhance the sample size by including patients from 2015 to nowadays?**

We agree with your comment. We could not extend the enrollment period due to financial reasons and human resources. Further study with a larger sample size is warranted to confirm our results.

3. **I suggest that genomic data and frequencies are compared with 1000 genomes projects, which gives a different % for the analysed SNPs in the East Assian population.**

We changed the genome frequencies to those of the 1000 Genomes Project in Table 1 and in the Results (page 8, lines 170–172). Reference 21 was changed to cite the 1000 Genomes Project.

4. **Moreover, when first used, the abbreviation of DAPT should appear.**

We added the word “DAPT” at its first appearance (page 4, line 66).

5. **Line 174-178. The sentence should be rephrased, too long and difficult to follow.**

We have modified the sentence to provide a better understanding for the readers (page 10, lines 175–180).

- We have added Ethics statement at the beginning of the Materials and Methods section (page 5, lines 96–100).
